# Residual feed intake in beef cattle and its association with carcass traits, ruminal solid-fraction bacteria, and epithelium gene expression

**DOI:** 10.1186/s40104-018-0283-8

**Published:** 2018-09-24

**Authors:** Ahmed A. Elolimy, Mohamed K. Abdelmegeid, Joshua C. McCann, Daniel W. Shike, Juan J. Loor

**Affiliations:** 1 Mammalian NutriPhysioGenomics, Department of Animal Sciences, University of Illinois, Urbana, IL USA; 2 Department of Animal Sciences, University of Illinois, Urbana, IL USA; 3 Faculty of Veterinary Medicine, Kafrelsheikh University, Kafr El-Shaikh, 33516 Egypt; 4 Division of Nutritional Sciences, Illinois Informatics Institute, University of Illinois, Urbana, IL USA

**Keywords:** Beef cattle, Carcass, Gene expression, Growth, RFI, Ruminal bacteria, Ruminal epithelium, Sex

## Abstract

**Background:**

Residual feed intake (RFI) describes an animal’s feed efficiency independent of growth performance. The objective of this study was to determine differences in growth performance, carcass traits, major bacteria attached to ruminal solids-fraction, and ruminal epithelium gene expression between the most-efficient and the least-efficient beef cattle. One-hundred and forty-nine Red Angus cattle were allocated to three contemporary groups according to sex and herd origin. Animals were fed a finishing diet in confinement for 70 d to determine the RFI category for each. Within each group, the two most-efficient (*n* = 6; RFI coefficient = − 2.69 ± 0.58 kg dry matter intake (DMI)/d) and the two least-efficient animals (*n* = 6; RFI coefficient = 3.08 ± 0.55 kg DMI/d) were selected. Immediately after slaughter, ruminal solids-fraction and ruminal epithelium were collected for bacteria relative abundance and epithelial gene expression analyses, respectively, using real-time PCR.

**Results:**

The most-efficient animals consumed less feed (*P* = 0.01; 5.03 kg less DMI/d) compared with the least-efficient animals. No differences (*P* > 0.10) in initial body weight (BW), final BW, and average daily gain (ADG) were observed between the two RFI classes. There were no significant RFI × sex effects (*P* > 0.10) on growth performance. Compared with the least-efficient group, hot carcass weight (HCW), ribeye area (REA), and kidney, pelvic, and heart fat (KPH) were greater (*P* ≤ 0.05) in the most-efficient cattle. No RFI × sex effect (*P* > 0.10) for carcass traits was detected between RFI groups. Of the 10 bacterial species evaluated, the most-efficient compared with least efficient cattle had greater (*P* ≤ 0.05) relative abundance of *Eubacterium ruminantium*, *Fibrobacter succinogenes*, and *Megasphaera elsdenii*, and lower (*P* ≤ 0.05) *Succinimonas amylolytica* and total bacterial density. No RFI × sex effect on ruminal bacteria was detected between RFI groups. Of the 34 genes evaluated in ruminal epithelium, the most-efficient cattle had greater (*P* ≤ 0.05) abundance of genes involved in VFA absorption, metabolism, ketogenesis, and immune/inflammation-response. The RFI × sex interactions indicated that responses in gene expression between RFI groups were due to differences in sex. Steers in the most-efficient compared with least-efficient group had greater (*P* ≤ 0.05) expression of *SLC9A1*, *HIF1A*, and *ACO2*. The most-efficient compared with least-efficient heifers had greater (*P* ≤ 0.05) mRNA expression of *BDH1* and lower expression (*P* ≤ 0.05) of *SLC9A2* and *PDHA1*.

**Conclusions:**

The present study revealed that greater feed efficiency in beef cattle is associated with differences in bacterial species and transcriptional adaptations in the ruminal epithelium that might enhance nutrient delivery and utilization by tissues. The lack of RFI × sex interaction for growth performance and carcass traits indicates that sex may not play a major role in improving these phenotypes in superior RFI beef cattle. However, it is important to note that this result should not be considered a solid biomarker of efficient beef cattle prior to further examination due to the limited number of heifers compared with steers used in the study.

**Electronic supplementary material:**

The online version of this article (10.1186/s40104-018-0283-8) contains supplementary material, which is available to authorized users.

## Background

The concept of residual feed intake (RFI), which is a commonly used measure for the efficiency of feed utilization in cattle independent from body weight (BW) and the level of production, was proposed more than 50 years ago by Koch et al. [1]. RFI is defined as the difference between the actual dry matter intake (DMI) of an animal and the expected DMI required for maintenance and growth estimated through a regression equation involving metabolic BW and average daily gain (ADG) [1]. The primary advantage of identifying the most-efficient animals (i.e., with low RFI) is to reduce DMI in beef systems without compromising growth performance because feed-related costs represent the largest production expense in beef production [2]. Any reduction in DMI to produce a unit of beef product would minimize feed costs, resulting in maximizing the overall profitability of beef industry.

A previous study indicated that ~ 20% of RFI variation in beef cattle could be explained by differences in rumen-related functions such as microbial digestion and epithelial metabolism [3]. Evidence indicates that efficient beef bulls and heifers have greater digestibility for dry matter (DM), organic matter (OM), neutral detergent fiber (NDF), protein, and total digestible nutrients (TDN) [4, 5], underscoring the vital role of ruminal microbes and epithelial tissue as key drivers of divergence in RFI. Thus, additional work to evaluate ruminal microbes and epithelium tissue between divergent RFI classes should help in identifying potential physiological mechanisms to enhance nutrient uptake and production efficiency in the most-efficient cattle.

The association between changes in ruminal bacteria profile and RFI divergence has been suggested previously. For example, compared with the least-efficient, the most-efficient Brahman bulls had greater abundance of *Bacteroidales* but lower *Prevotella* [6]. In a recent study, Liang et al. [7] reported that the most-efficient lambs had lower *Butyrivibrio fibrisolvens* and *Escherichia coli*. The association between feed efficiency in beef cattle and changes in ruminal epithelium gene transcription was addressed recently. Kong et al. [8] reported that, compared with the least-efficient, the most-efficient finishing steers had greater expression of genes involved in VFA absorption (dynamin-2 (*DNM2);* tubulin beta-5 (*TUBB5*); tubulin alpha-4a (*TUBA4A*)). A greater expression of genes involved in cell growth and proliferation such as solute carrier family 26 member 3 (*SLC26A3*), caveolin 1 (*CAV1*), NAD(P)H quinone dehydrogenase 1 (*NQO1*), and regulator of G protein signaling 5 (*RGS5*) was detected in steers classified as high gain-low feed intake compared with least-efficient steers [9].

Most of the available RFI studies in beef cattle are limited to either steers or heifers, hence, the association between RFI and sex also warrants further investigation. It was hypothesized that holistic evaluation of the relationship between RFI and ruminal function, including ruminal bacteria and epithelial responses, in both steers and heifers would provide insights into biological mechanisms underpinning variation in RFI divergence between sexes. Such data would also help to uncover biomarkers for identifying the most-efficient animals. We addressed these issues using Red Angus beef steers and heifers classified as most- or least-efficient during a 70-d finishing period. Major ruminal bacteria that play key roles in cellulose digestion (*Fibrobacter succinogenes, Eubacterium ruminantium*) [10, 11], hemicellulose breakdown (*Prevotella bryantii*) [12], xylan degradation (*Butyrivibrio proteoclasticus*) [13], starch consumption (*Succinimonas amylolytica, Streptococcus bovis, Succinovibrio dextrinosolvens*) [14], lactate utilization (*Megasphaera elsdenii*, *Selenomonas ruminantium*) [15, 16], and ruminal biohydrogenation (*Anaerovibrio lipolytica*) [17] were quantified in the rumen-solids fraction. Additionally, the abundance of rumen epithelial genes playing key roles in VFA absorption [18], metabolism [19, 20], ketogenesis [21, 22], and immune/inflammation-response [23] that could be affected by the changes in feed intake between RFI groups were measured at slaughter.

## Methods

All the procedures for this study were approved by Institutional Animal Care and Use Committee of the University of Illinois under protocol number 12009.

### Animals, experimental design, and diets

Three contemporary groups of Red Angus cattle (Group 1: 35 steers; Group 2: 37 heifers; Group 3: 80 steers) were used. Animal BW was recorded on 2 consecutive days at the beginning of the RFI test and averaged to determine initial BW. Animals were blocked in group pens based on their initial BW to limit domination. Each pen was equipped with GrowSafe® individual feed intake system (GrowSafe Systems Ltd., Alberta, Canada) allowing for individual intake data. Upon blocking the animals, electronic identification tags were attached to the left ear to help in tracking individual feed intake. The experimental period had a duration of 70 d (21 d of adaptation to traditional mid-west finishing feedlot diet and environment + 70 d of data collection). The experimental diet was formulated to meet all nutrient requirements for growing cattle according to NRC (Table 1). The experimental diet was fed in excess to achieve on average 5% of refusal daily. It was offered twice daily and all animals had ad libitum access to feed and water throughout the experimental period.

**Table 1:** Diet and nutrient composition fed to finishing cattle on RFI testing for 70 d

Items	Inclusion, % DM
Ingredient, %	
High-moisture corn	20.00
Cracked corn	40.00
Corn silage	20.00
DDGS^1^	10.00
Supplement, % of DM	10.00
Ground corn	76.19
Urea	5.99
Limestone	15.89
Dairy trace mineral sal^t2^	0.91
Rumensin 90^3^	0.15
Tylan 40^4^	0.10
Vitamin A-V blend	0.77
Analyzed nutrient content, %	
CP	15.62
NDF	20.82
ADF	8.17
Fat	3.71

^1^Dried distillers grains with solubles

^2^Contained 8.5% Ca (as CaCO_3_), 5% Mg (as MgO and MgSO_4_), 7.6% K (as KCl), 6.7% Cl (as KCl), 10% S (as S_8_, prilled), 0.5% Cu (as CuSO_4_ and Availa-4; Zinpro Performance Minerals; Zinpro Corp, Eden Prairie, MN)

^3^Elanco Animal Health, Greenfield, IN

^4^Elanco Animal Health, Greenfield, IN

### Animal feedlot test and RFI calculation

The BW, ADG, midpoint metabolic weight (MMW), and DMI were calculated for each animal as described previously [24]. Briefly, all animals were weighed at the beginning, end and at every 14 d of the experimental period prior to feeding to minimize differences in gut fill but with no food and water restriction. The ADG was estimated as the slope of the linear regression between BW and days on feed. Midpoint metabolic weight was calculated as midpoint BW^0.75^, with midpoint BW computed as the sum of the initial BW and the product of ADG multiplied by half of the days on RFI test. The DMI for each animal was recorded daily via the GrowSafe® system. After the end of the collection of feed intake data, cattle had access to the same diet ad libitum and remained in the same pens for 30 d until reaching 1.15 cm backfat depth measured by ultrasound.

Animals were slaughtered after a 16-h fast. Hot carcass weight (HCW) was recorded, and kidney, pelvic, and heart fat (KPH), ribeye area (REA), backfat thickness (BFT), and marbling score (100 = practically devoid, 200 = traces, 300 = slight, 400 = small, 500 = modest, 600 = moderate, 700 = slightly abundant, 800 = moderately abundant) collected after a 24-h chill at − 4 °C. Amount of boneless, closely-trimmed retail cuts from the high-value parts of the carcass (round, loin, rib, and chuck), i.e. USDA yield grade (YG), was calculated by evaluating the amount of HCW, KPH, BFT, and REA. Growth performance and carcass traits are shown in Table 2.

**Table 2:** Growth and carcass traits of the most- (*n* = 6) and the least- (*n* = 6) efficient beef cattle on RFI testing for 70 d during the finishing period

Items	RFI^1^		SE^2^	Sex		SE^3^	*P*-value		
	Most-efficient animals	Least-efficient animals		Steers	Heifers		RFI	Sex	RFI × sex
Growth performance									
Initial BW, kg	814.25	836.06	45.95	847.31	803.00	53.05	0.75	0.52	0.87
Final BW, kg	1124.25	1173.63	64.77	1189.88	1108.00	74.79	0.61	0.40	0.59
MMW^4^, kg^0.75^	173.06	166.39	5.93	179.50	159.95	6.85	0.45	0.05	0.43
ADG, kg/d	1.83	1.77	0.13	2.02^a^	1.57^b^	0.15	0.75	0.04	0.91
DMI, kg/d	18.36^b^	23.39^a^	1.13	22.62	19.13	1.24	0.01	0.06	0.77
RFI coefficient, kg/d	-2.69^b^	3.08^a^	0.58	0.35	0.043	0.63	< 0.01	0.71	0.52
Carcass traits									
HCW^5^, kg	357.56^a^	323.53^b^	9.61	367.26^a^	313.83^b^	10.52	0.04	< 0.01	0.66
KPH^6^, %	2.69^a^	2.25^b^	0.14	2.06^b^	2.88^a^	0.14	0.05	< 0.01	0.33
REA^7^, cm^2^	89.40^a^	78.52^b^	3.20	85.17	82.74	3.51	0.04	0.58	0.62
BFT^8^, mm	12.19	11.81	1.58	11.81	12.19	1.82	0.87	0.87	0.48
Marbling score^9^	457.5	442.5	20.63	407.5^b^	492.5^a^	23.82	0.62	0.02	0.21
YG^10^	2.45	2.97	0.34	2.87	2.56	0.39	0.32	0.54	0.73

Means with different superscripts (^a, b^) within row represent differences among overall RFI or sex effects (*P* ≤ 0.05)

^1^*RFI* residual feed intake

^2^*SE* standard error of the mean for RFI effect

^3^*SE* standard error of the mean for sex effect

^4^*MMW* midpoint metabolic weight

^5^*HCW* hot carcass weight

^6^*KPH* kidney, pelvic, and heart

^7^*REA* ribeye area

^8^*BFT* backfat thickness

^9^For marbling score 100 = practically devoid, 200 = traces, 300 = slight, 400 = small, 500 = modest, 600 = moderate, 700 = slightly abundant, 800 = moderately abundant

^10^*YG* yield grade = 2.5 + 0.984 × BFT (cm) + 0.20 × KPH (%) + 0.0084 × HCW (kg) − 0.0497 × LM area (cm^2^) [67]

### Ruminal digesta and tissue sampling

After slaughter, the rumen from the most- and the least-efficient animals was immediately removed and sampling of the solids-fraction and epithelium performed. Mixed ruminal contents were collected and squeezed through 4-layers of cheesecloth allowing the separation of solids from the liquid fraction before sampling and placing in 50 mL sterilized tubes. The ruminal rumen papillae was excised (approximately 300 mg) from the central region of the ventral sac [8], and immediately washed with PBS before placing in 1.5 mL sterilized tubes. All samples were immediately snap-frozen in liquid nitrogen, transported to the laboratory. Rumen solids-fraction was stored at − 20 °C and ruminal epithelium stored at − 80 °C until further analysis.

### Ruminal bacteria DNA extraction and the amplification of 16S rRNA genes

The procedure for extraction of bacteria attached to the solids-fraction was as previously described [25]. Briefly, 25 g of sample was added into 100 mL of chilled extraction buffer composed of 100 mmol/L Tris/HCl, 10 mmol/L EDTA, 0.15 mol/L NaCl at pH of 8.0. The mixture was homogenized by polytron (Kinematica Inc., Bohemia, NY, USA) for 2 min, then centrifuged at 500 × *g* for 15 min at 4 °C to keep bacterial cells in the supernatant. The resulting supernatant was centrifuged at 10,000 × *g* for 25 min at 4 °C. The pellet was harvested, freeze-dried, and stored at − 20 °C. Twenty-five mg of the pellet was used to isolate total genomic DNA using the repeated bead-beating plus column method described by Yu and Morrison [26] for mechanical lysis of bacterial cell wall employing the QIAamp DNA mini kit (QIAGEN, CA, USA) for DNA purification. The DNA quantity and quality were checked using 0.8% (*wt/v*) agarose gel electrophoresis and NanoDrop spectrophotometer (ND 1000, NanoDrop Technologies, Inc., Wilmington, DE, USA) at 260 nm. Extracted DNA was standardized to 8 ng/μL for quantitative PCR (qPCR) reactions.

Primers were selected to amplify 10 of major ruminal bacteria species play key roles in cellulose and hemicellulose digestion, xylan degradation, proteolysis, propionate production, lactate utilization and ruminal biohydrogenation [27], as listed in Additional file 1. A total of 10 μL of qPCR mixture contained 4 μL sample DNA, 5 μL 1× SYBR Green with ROX (Quanta BioSciences, Gaithersburg, MD, USA), 0.4 μL each of 10 μmol/L forward and reverse primers, and 0.2 μL DNase/RNase free water in a MicroAmpTM Optical 384-Well Reaction Plate (Applied Biosystems, Foster City, CA, USA). Negative controls without template DNA and samples were run on the same plate in triplicate. The qPCR reactions were performed with ABI PRISM 7900HT Sequence Detection System (Applied Biosystems, Foster City, CA, USA) using the following program: initial denaturation at 95 °C for 5 min, followed by 40 cycles of 1 s at 95 °C and 30 s annealing at 60 °C. A final dissociation stage was performed to determine the specificity of the amplification. Relative abundance of bacterial species, including *Anaerovibrio lipolytica*, *Butyrivibrio proteoclasticus*, *Eubacterium ruminantium*, *Fibrobacter succinogenes*, *Megaspheara elsdenii*, *Prevotella bryantii*, *Selenomonas ruminantium*, *Succinimonas amylolytica*, *Streptococcus bovis*, and *Succinivibrio dextrinosolvens*, was calculated using the geometric mean of two universal primers of bacteria general 1 and bacteria general 2 [28, 29] (Additional file 1) with the efficiency-corrected Δ^−CT^ method [30]. The copy number of total bacterial 16S rRNA genes in rumen solids-fraction was measured to estimate the total bacterial density using qPCR with the bacteria general 3 (Additional file 1) which was commercially synthesized (IDT, Coralville, IA, USA) to target the universal bacteria, following a procedure described previously [31].

### Ruminal epithelium RNA extraction and cDNA synthesis

Ten mg tissue was immediately placed in 1.2 mL QIAzol Lysis Reagent (Qiagen, Valencia, CA, USA) and homogenized with 5 mm stainless steel beads using a Mini-Beadbeater (BioSpec Products, Bartlesville, OK, USA) with two 30 s cycles, and 1 min incubation on ice in between the cycles. The sample was then centrifuged for 10 min at 12,000 × *g* and 4 °C, and the supernatant transferred to a separate tube and mixed with 240 μL of chloroform. After centrifugation for 15 min at 12,000 × *g* at 4 °C, the aqueous phase was transferred to a fresh 1.5 centrifuge tube, mixed with 900 μL of 100% ethanol (Decon Laboratories, Inc., King of Prussia, PA, USA). The extracted RNA was cleaned using miRNeasy mini kit columns (Qiagen, Valencia, CA, USA) following the manufacturer’s instructions. All samples were treated with DNaseI (Qiagen, Valencia, CA, USA) to remove genomic DNA. Total RNA quantification was determined using a Nanodrop ND-1000 (Nanodrop Technologies, Rockland, DE, USA). The purity and integrity of extracted RNA was evaluated using a Fragment Analyzer (Advanced Analytical, Ames, IA, USA) with an average RIN score of 8.8 (minimum RIN = 7.4). The RNA was diluted to 100 ng/μL with DNase/RNase-free water. For cDNA synthesis, a mixture of 4 μL of diluted RNA, 5 μL of Random Primers (3 μg/μL; Roche Diagnostics, Basel, Switzerland), and 45 μL of DNase/RNase-free water was incubated at 65 °C for 5 min and kept on ice for 3 min.

A total of 36 μL of master mix composed of 16 μL 5× Reaction Buffer (Thermo Fisher Scientific, Waltham, MA, USA), 4 μL of Oligo dT18 (Custom DNA Oligo Tubes, Integrated DNA Technologies, Coralville, IA, USA), 1 μL of RevertAid Reverse Transcriptase (Thermo Fisher Scientific, Waltham, MA, USA), 8 μL of 10 mmol/L dNTP mix (Invitrogen, Carlsbad, CA, USA), 0.5 μL of RNase Inhibitor (Thermo Fisher Scientific, Waltham, MA, USA), and 6.5 μL of DNase/RNase-free water, was added. The reaction was performed in an Eppendorf Mastercycler® Gradient using the following temperature program: 25 °C for 5 min, 42 °C for 60 min, and 70 °C for 5 min. The cDNA was then diluted 1:4 with DNase/RNase-free water, prior to quantitative reverse transcription-PCR (qRT-PCR) analysis.

### Quantitative PCR

Complete information about primer sequences and qRT-PCR performance are reported in Additional file 1. Their PCR products were verified using gel electrophoresis and sequencing (data not shown). The qRT-PCR reaction components and instrument conditions were the same as described for bacterial qPCR. All reactions were run in triplicate. A six-point relative standard curve was used to determine gene expression [23]. The most-concentrated standard (100 ng/μL) was prepared by combining 30 μL from all samples after converting RNA into cDNA. Using molecular grade water, the subsequent standards were prepared through 1:4 serial dilutions of the most-concentrated standard to eventually get standard 1, 2, 3, 4, 5, and 6 contain 100, 25, 6.250, 1.560, 0.391, and 0.098 ng/μL, respectively, for the standard curve. After the completion of qPCR, Ct value of each sample has been used to calculate the cDNA quantity through the standard curve. Relative quantities were calculated using the geometric mean of previously-validated internal control for work with ruminal epithelium: CKLF like MARVEL transmembrane domain containing 6 (*CMTM6*), ELKS/RAB6-interacting/CAST family member 1 (*ERC1*), and mitochondrial ribosomal protein L39 (*MRPL39*) [20, 32] with the efficiency-corrected 2^−ΔΔCt^ method [33]. The gene expression results reported in Table 1 are the log_2_ back-transformed LSM and standard error.

### Statistical analysis

The RFI was calculated using the PROC MIXED procedure of SAS procedure of SAS 9.4 (SAS Institute Inc., Cary, NC, USA). Animals were separated into 3 contemporary groups using sex and source of origin. RFI was calculated within contemporary group, and assumed to represent the residuals from a multiple regression model regressing DMI on ADG, MMW, and BFT, using pen as a random effect in the following model:

Expected DMI = *β*_0_ + *β*_1_ × ADG + *β*_2_ × MMW + *β*_3_ × BFT + *β*_4_ + ɛ.

in which *β*_0_ is the *y*-intercept, *β*_1_ is the partial regression coefficient of ADG, *β*_2_ is the partial regression coefficient of MMW, *β*_3_ is the partial regression coefficient of BFT, *β*_4_ is the random effect of pen and ɛ is the error term. The coefficient of determination (*R*^2^) was 0.76, 0.65, and 0.42 for group 1, 2, and 3, respectively. As proposed by Basarab et al. [24], the RFI (kg DMI/d) for each individual animal was then calculated as the difference between the daily DMI and the expected DMI. All animals were ranked by RFI, then the two most extreme low and high RFI animals from each group were selected to form two RFI groups: the most-efficient animals (*n* = 6) and the least-efficient animals (*n* = 6), each composed of 6 extreme animals. Individual animal was the experimental unit, and dependent variables included growth performance, carcass traits, relative abundance of bacteria, and ruminal epithelium genes. Model included the fixed effects of RFI category, sex, and RFI × sex. Separation of LSM for significant effects was accomplished using the Tukey’s option within the MIXED procedure of SAS. Logit transformation (*z* = log[*p*/(1-*p*)]) was applied for bacterial abundance to ensure normal distribution of the data, where *p* represents the relative abundance of a bacterial species. Genes were log_2_ transformed before statistical analysis but the data were back-transformed for presentation in tables and figures. Significance was declared at *P* ≤ 0.05, and trends toward significance were discussed at 0.05 < *P* ≤ 0.10.

## Results

### Animal performance and carcass traits

The mean difference in RFI between the most- and least-efficient animals was 5.77 kg DMI/d. The most-efficient animals consumed 2.69 ± 1.56 kg DMI/d less feed, whereas the least-efficient animals consumed 3.08 ± 1.56 kg DMI/d more feed than expected. The most-efficient group had lower (*P* = 0.01) DMI than least-efficient counterparts. No differences (*P* > 0.10) for initial BW, final BW, MMW, and ADG were observed between RFI groups (Table 2). There were no significant effect (*P* > 0.10) for the interaction of RFI and sex on growth performance (Table 2). For the sex effect on growth traits, steers had greater MMW (*P* = 0.05) and ADG (*P* = 0.04) and tended to have greater DMI (*P* = 0.06) than heifers. No RFI × sex interactions (*P* > 0.10) were detected between RFI classes in steers or heifers (Table 2).

A main effect of RFI grouping was detected in carcass traits due to greater (*P* ≤ 0.05) HCW, KPH, and REA in the most-efficient compared with least-efficient animals (Table 2). Regarding the RFI × sex effect on carcass traits, no significant differences (*P* > 0.10) were detected (Table 2). With respect to sex effects on carcass traits, there were significant differences between steers and heifers, with steers having greater HCW but lower (*P* ≤ 0.05) KPH and marbling score (Table 2).

### Ruminal bacteria

Among target bacteria, *S. ruminantium* and *S. dextrinosolvens* were the most abundant averaging 0.12% and 0.11% of the 16S rRNA copy numbers. There was no significant (*P* > 0.10) effect of RFI or sex on *A. lipolytica*, *B. proteoclasticus*, *P. bryantii*, *S. ruminantium*, *S. bovis*, and *S. dextrinosolvens*. The most-efficient cattle had greater (*P* ≤ 0.05) abundance of *E. ruminantium* (218.4%), *F. succinogenes* (290.0%), and *M. elsdenii* (233.3%), and lower abundance (*P* < 0.01) of *S. amylolytica* (− 81.6%). The lower 16S rRNA gene copy numbers/ng DNA indicated that total ruminal bacteria abundance was lower (*P* = 0.05) in the most-efficient animals (Table 3). There was no RFI × sex effect (*P* > 0.10) on the relative abundance of the selected bacteria or total bacteria density. For the sex effect during the same time-frame, steers compared with heifers had greater relative abundance of *E. ruminantium* and *F. succinogenes* (Table 3).

**Table 3:** Relative abundance (%) of 10 targeted rumen bacteria species and the 16S rRNA gene copy numbers of the total rumen bacterial community in the rumen solids-fraction of the most- (*n* = 6) and the least- (*n* = 6) efficient beef cattle on RFI testing for 70 d during the finishing period

Items^1^	RFI^2^		SE^3^	Sex		SE^4^	*P*-value		
	Most-efficient animals	Least-efficient animals		Steers	Heifers		RFI	Sex	RFI × sex
Target bacterial species									
*A. lipolytica*	1.80 × 10^−4^	9.00 × 10^−5^	0.27	1.20 × 10^−4^	1.30 × 10^− 4^	0.31	0.48	0.96	0.45
*B. proteoclasticus*	1.91 × 10^−2^	1.12 × 10^− 2^	0.22	1.33 × 10^− 2^	1.60 × 10^− 2^	0.25	0.47	0.80	0.66
*E. ruminantium*	9.13 × 10^-2a^	4.18 × 10^-2b^	0.10	9.09 × 10^-2a^	4.20 × 10^-2b^	0.11	0.05	0.05	0.84
*F. succinogenes*	2.90 × 10^-4a^	1.00 × 10^-4b^	0.13	3.00 × 10^-4a^	0.90 × 10^-4b^	0.14	0.03	0.02	0.79
*M. elsdenii*	7.00 × 10^-5a^	3.00 × 10^-5b^	0.12	4.00 × 10^−5^	5.00 × 10^−5^	0.12	0.04	0.73	0.35
*P. bryantii*	4.31 × 10^−2^	3.52 × 10^−2^	0.14	5.37 × 10^−2^	2.82 × 10^− 2^	0.16	0.67	0.20	0.40
*S. ruminantium*	1.25 × 10^−1^	1.17 × 10^−1^	0.08	1.21 × 10^−1^	1.20 × 10^−1^	0.09	0.80	0.97	0.95
*S. amylolytica*	0.70 × 10^-4b^	3.80 × 10^-4a^	0.14	2.40 × 10^−4^	1.20 × 10^−4^	0.14	< 0.01	0.16	0.83
*S. bovis*	5.63 × 10^−3^	6.75 × 10^−3^	0.25	6.30 × 10^− 3^	6.03 × 10^− 3^	0.29	0.83	0.96	0.48
*S. dextrinosolvens*	4.81 × 10^−2^	1.22 × 10^−2^	0.48	1.38 × 10^− 2^	4.26 × 10^− 2^	0.56	0.41	0.49	0.28
Bacteria density									
The 16S rRNA gene copy numbers (log_10_)/ng DNA^5^	8.03^b^	8.07^a^	0.01	8.05	8.05	0.01	0.05	0.89	0.60

Means with different superscripts (^a, b^) within row represent differences among overall RFI or sex effects (*P* ≤ 0.05)

^1^Data were logit transformed to ensure normality of residuals

^2^*RFI* residual feed intake

^3^*SE* standard error of the mean for RFI effect

^4^*SE* standard error of the mean for sex effect

^5^16S rRNA gene copy number (log_10_)/ng DNA

### Epithelium gene expression

#### VFA absorption

**Fig. 1:**
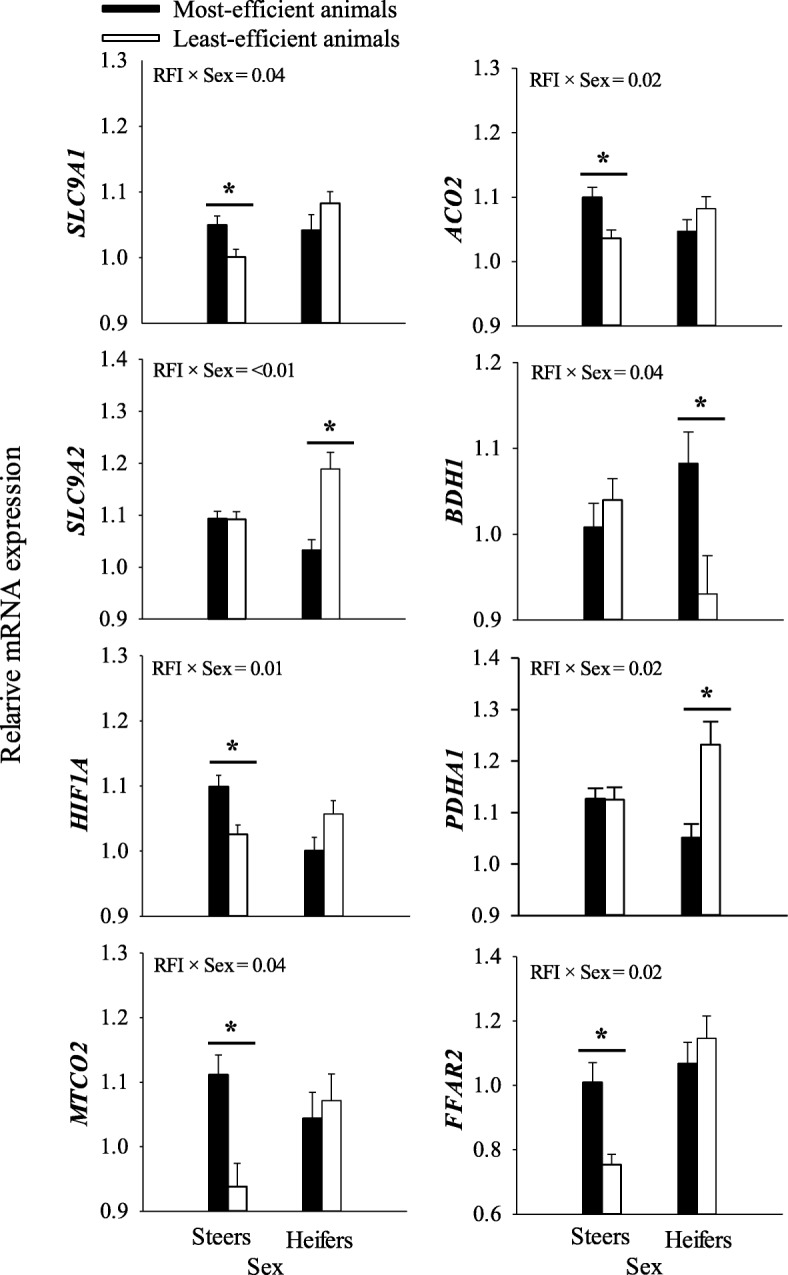
Significant RFI and sex interactions (*P ≤ 0.05) on relative mRNA expression of rumen epithelium genes in the most- (*n* = 6) and the least- (*n* = 6) efficient beef cattle on RFI testing for 70 d during the finishing period

Compared with the least-efficient, the most-efficient animals had greater (*P* = 0.02) solute carrier family 16 member 3 (*SLC16A3*) expression and tended to have greater (*P* = 0.06) solute carrier family 26 member 3 (*SLC26A3*) expression (Table 4). In contrast, the most-efficient cattle had lower (*P* = 0.01) solute carrier family 9 member A2 (*SLC9A2*) expression (Table 4). An RFI × sex effect (*P* ≤ 0.05) was also detected for solute carrier family 9 member A1 (*SLC9A1*) and hypoxia inducible factor 1 alpha subunit (*HIF1A*) because of greater expression in the most-efficient compared with least-efficient steers, whereas no significant differences (*P* > 0.10) for both genes were detected between RFI classes for heifers (Fig. 1). For the sex effect, compared with heifers, steers tended (*P* = 0.08) to have lower expression of *SLC9A1* (Table 4).

**Table 4:** Relative mRNA expression of rumen epithelium genes in the most- (*n* = 6) and the least- (*n* = 6) efficient beef cattle on RFI testing for 70 d during the finishing period

Items	RFI^1^		SE^2^	Sex		SE^3^	*P*-value		
	Most-efficient animals	Least-efficient animals		Steers	Heifers		RFI	Sex	RFI × sex
VFA absorption									
*SLC16A1*	1.06	0.96	0.04	1.05	0.97	0.05	0.13	0.23	0.43
*SLC16A3*	1.18^a^	1.04^b^	0.03	1.11	1.11	0.03	0.02	0.89	0.96
*SLC26A3*	1.00	0.81	0.06	0.98	0.83	0.07	0.06	0.11	0.75
*HCAR1*	1.08	1.12	0.05	1.07	1.13	0.06	0.65	0.47	0.18
*SLC9A1*	1.05	1.04	0.01	1.03	1.06	0.01	0.81	0.08	0.04
*SLC9A2*	1.06^b^	1.14^a^	0.02	1.09	1.11	0.02	0.01	0.48	< 0.01
*SLC9A3*	1.09	1.11	0.05	1.13	1.07	0.05	0.74	0.44	0.12
*APPBP2*	1.01	1.03	0.04	1.01	1.02	0.04	0.80	0.83	0.11
*HIF1A*	1.05	1.04	0.01	1.06	1.03	0.01	0.69	0.12	0.01
VFA metabolism									
*ACSS1*	1.12	1.23	0.14	1.25	1.10	0.15	0.57	0.47	0.56
*ACSS2*	0.99	0.95	0.05	0.98	0.96	0.06	0.54	0.80	0.60
*ACO1*	0.69	0.69	0.02	0.70	0.68	0.02	0.95	0.62	0.08
*ACO2*	1.07	1.06	0.01	1.07	1.06	0.01	0.42	0.86	0.02
*PCCA*	1.04	1.01	0.03	0.99	1.06	0.03	0.32	0.07	0.73
*SLC25A20*	1.11	1.05	0.03	1.12^a^	1.04^b^	0.03	0.11	0.04	0.17
Ketogenesis									
*ACADS*	1.05	1.00	0.02	1.01	1.04	0.03	0.12	0.41	0.33
*ACAT1*	1.17	1.06	0.04	1.13	1.09	0.05	0.09	0.46	0.45
*HMGCL*	1.10^a^	1.01^b^	0.03	1.04	1.06	0.03	0.04	0.53	0.44
*BDH1*	1.04	0.98	0.03	1.02	1.00	0.03	0.13	0.59	0.04
*HMGCS2*	1.16^a^	0.99^b^	0.06	1.11	1.04	0.05	0.04	0.37	0.47
*TECR*	1.05	1.04	0.02	1.05	1.04	0.03	0.83	0.60	0.08
Pyruvate Metabolism									
*LDHA*	1.03^a^	0.97^b^	0.02	0.98	1.02	0.02	0.02	0.09	0.96
*LDHB*	1.07	0.99	0.03	1.00	1.06	0.04	0.09	0.18	0.83
*PDHA1*	1.09^b^	1.18^a^	0.02	1.13	1.14	0.03	0.02	0.70	0.02
*PC*	1.04	1.02	0.03	1.01	1.06	0.03	0.74	0.20	0.45
Other metabolic pathways									
*PPARA*	1.27	1.25	0.05	1.25	1.27	0.06	0.80	0.85	0.79
*PPARG*	1.00	1.00	0.02	0.98	1.02	0.02	0.88	0.14	0.66
*PPARD*	1.07^a^	0.99^b^	0.02	1.01	1.04	0.02	0.01	0.18	0.37
*FFAR2*	1.04	0.93	0.04	0.87^b^	1.11^a^	0.05	0.09	< 0.01	0.02
*RGS5*	1.02	1.06	0.09	0.99	1.10	0.11	0.75	0.38	0.51
*NQO1*	1.14	1.04	0.04	1.06	1.11	0.05	0.12	0.41	0.80
Immune/inflammation-response									
*TLR2*	1.02	0.97	0.02	0.98	1.01	0.03	0.15	0.41	0.18
*TLR4*	1.09	1.15	0.07	1.09	1.14	0.08	0.56	0.57	0.68

Means with different superscripts (^a, b^) within row represent differences among overall RFI or sex effects (*P* ≤ 0.05)

^1^*RFI* residual feed intake

^2^*SE* standard error of the mean for RFI effect

^3^*SE* standard error of the mean for sex effect

#### VFA metabolism

Among the 6 genes measured for VFA metabolism, none was significantly affected by RFI classification (*P* > 0.10) (Table 4). Compared with the least-efficient steers, the most-efficient steers had greater (*P* = 0.02) aconitase 2 (*ACO2*) expression. For the sex effect, compared with heifers, steers had greater solute carrier family 25 member 20 (*SLC25A20*) (*P* = 0.04) and tended to have lower expression (*P* = 0.07) of propionyl-CoA carboxylase alpha subunit (*PCCA*).

#### Ketogenesis

Compared with the least-efficient, the most-efficient animals had greater (*P* ≤ 0.05) mRNA expression of 3-hydroxymethyl-3-methylglutaryl-CoA lyase (*HMGCL*) and 3-hydroxy-3-methylglutaryl-CoA synthase 2 (*HMGCS2*), whereas acetyl-CoA acetyltransferase 1 (*ACAT1*) tended to be greater (*P* = 0.09) (Table 4). Sex had no effect on the ketogenic genes (*P* > 0.10). For the RFI × sex interaction, the most-efficient heifers had greater (*P* = 0.04) 3-hydroxybutyrate dehydrogenase 1 (*BDH1*) expression compared with the least-efficient heifers.

#### Pyruvate metabolism

Compared with the least-efficient, the most-efficient animals had greater (*P* = 0.02) lactate dehydrogenase A (*LDHA*) expression and tended to have greater (*P* = 0.09) lactate dehydrogenase B (*LDHB*), whereas the expression of pyruvate dehydrogenase alpha 1 (*PDHA1)* was lower (*P* = 0.02) (Table 4). For the RFI × sex interaction, the most-efficient heifers had lower (*P* = 0.02) *PDHA1* expression compared with the least-efficient heifers.

#### Other metabolic pathways and immune response

Compared with the least-efficient, the most-efficient animals had greater (*P* = 0.01) expression of peroxisome proliferator activated receptor delta (*PPARD*) and tended to have greater (*P* = 0.09) expression of free fatty acid receptor 2 (*FFAR2*) (Table 4). For the sex effect, steers had lower (*P* ≤ 0.01) *FFAR2* in comparison with heifers (Table 4). For the RFI × sex interaction, the most-efficient steers had greater (*P* ≤ 0.05) expression of *FFAR2* compared with the least-efficient steers, but no differences were detected for *FFAR2* between RFI groups for heifers (*P* > 0.10). The expression of the immune-responsive toll like receptor 2 (*TLR2*) and toll like receptor 4 (*TLR4*) was not affected (*P* > 0.10) by RFI, sex, or RFI × sex interaction.

## Discussion

### Animal performance and carcass traits

Compared with the least-efficient animals, the greater HCW and REA observed in the most-efficient animals is in line with several studies investigating the relationship between RFI grouping and protein deposition in beef cattle [34, 35]. The similar marbling score and BFT between RFI groups was driven primarily by the lack of enough biological replicates as carcass composition was not the main objective of the study. Other studies found that marbling was greater in the least-efficient steers [36, 37]. Despite this, in the future, it will be important to determine with greater numbers of animals whether lower DMI in feed-efficient cattle is associated with differences in intramuscular fat. With that type of information we could better address the issue of whether the control of marbling in feed-efficient cattle occurs primarily at the tissue level.

### Ruminal bacteria

Several studies indicated that superior feed efficiency (RFI) in beef steers is associated with shifts in rumen bacterial composition in favor of improving feedstuff digestibility [38, 39], but none of those studies investigated whether sex, i.e. steers vs. heifers, would impact bacterial profile. The greater abundance of *F. succinogenes* (290%) and *E. ruminantium* (218%) in the most-efficient animals could have been associated with improvements in fiber degradation and feed digestibility. In the same line, Elolimy et al. [27] reported that the most-efficient dairy cows tended to have greater *F. succinogenes* around calving. *F. succinogenes,* a strictly anaerobic Gram-negative bacterium, is one of the most-important cellulose-degrading bacteria in the rumen, and ferments cellulose, xylan, cellobiose, and glucose to generate succinate, acetate and formate [40]. *E. ruminantium*, a Gram-positive bacteria, also plays a cellulolytic role in the rumen [41]. A better ability to ferment fiber through greater abundance *F. succinogenes* and *E. ruminantium* is supported by previous reports showing that the most-efficient beef bulls and heifers had greater rates of DM, OM, NDF, protein, and TDN digestibility [4, 5]. *M. elsdenii*, a Gram negative bacterium, utilizes lactate to produce butyrate and propionate by reverse β-oxidation [15]. Additionally, *E. ruminantium* produces butyrate through cellulose degradation [11]. In a recent study, Elolimy et al. [27] found that the most-efficient dairy cows tended to have greater *M. elsdenii* around calving. Therefore, the greater relative abundance of *M. elsdenii* and *E. ruminantium* in the most-efficient cattle in the current study could have led to increases in the molar proportion of butyrate and propionate in the rumen. In support of this speculation, Muya et al. [42] reported that neonatal dairy bulls and heifers receiving a 50-mL oral dose of *M. elsdenii* at 14 d of age had greater intraruminal butyrate production and plasma β-hydroxybutyrate (BHBA) at weaning (42 d of age).

Several lines of evidence indicate that the most-efficient cattle and sheep have greater butyrate, propionate, and propionate:acetate ratio in the rumen [7, 43]. Enhancing the production of butyrate and propionate in the rumen is of physiologic significance for increasing energy retention from the feed since butyrate is metabolized by the ruminal epithelium to BHBA, whereas propionate is the main precursor for hepatic gluconeogenesis [44, 45]. Thus, although ruminal VFA concentrations were not measured in this study, we speculate that the most-efficient animals may have had greater levels of butyrate and propionate partly in response to the greater abundance of *M. elsdenii* and *E. ruminantium* in the rumen. Due to its role in the removal of lactate, *M. elsdenii* also helps maintain ruminal pH and control lactic acidosis [46]. Several studies observed a dramatic decrease in cellulolytic bacteria such as *F. succinogenes, Ruminococcus albus,* and *Ruminococcus flavefaciens*, but an increase in amylolytic species, e.g. *S. amylolytica* and *Ruminobacter amylophilus*, under low ruminal pH conditions such as subacute ruminal acidosis (SARA) [14, 47]. Therefore, greater *M. elsdenii* abundance in the most-efficient cattle in the present study may have helped control ruminal pH, in turn facilitating the growth of cellulolytic *F. succinogenes* and *E. ruminantium* instead of amylolytic species such as *S. amylolytica*.

The discrepancy between a greater abundance of 3 important bacterial species and lower total bacterial population density in the most-efficient cattle could be partly attributed to the fact we only measured a small number of bacterial species. Therefore, future studies to elucidate how shifts in rumen microbiota and its fermentation patterns are associated with RFI divergence in finishing beef cattle are necessary. Because bacteria comprise 95% of the whole ruminal microbial community and contribute the most to feedstuff digestion, a lower density of total bacteria could be taken as indication that digestive function might be compromised in the most-efficient animals [48]. Despite that, other studies revealed that feed-efficient beef bulls and heifers had higher feed digestibility for DM, OM, NDF, protein, and TDN [4, 5]. This suggests that 16S gene copy numbers may not reflect the actual capacity of feed digestion in efficient cattle. The lower total bacterial density observed in the most-efficient cattle could have been attributed to lower DMI detected during the study since it is known that microbial growth is strongly correlated with the level of voluntary DMI [49]. Decreasing DMI would slow down the ruminal passage rate which is associated with increased energy costs of maintenance for microbes [50] leading to a decrease in the overall microbial population in feed-efficient cattle in the current study.

### Ruminal epithelium gene expression

#### VFA absorption and metabolism

Shifts in ruminal bacterial species in the most-efficient animals in the present study and the expected changes in VFA profile were possibly associated with alteration in the expression of various genes in ruminal epithelium. Expression of *SLC16A3*, *SLC26A3*, and *HIF1A* participate in the transepithelial absorption of VFA [18, 51]. Butyrate induced the transcription of *SLC16A3*, *SLC26A3*, and *HIF1A* in sheep ruminal epithelia, human breast cancer, and colon cell lines [52–54]. Therefore, the concurrent increase in the expression of those genes in the most-efficient animals might have been part of functional adaptations to enhance absorptive capacity in the most-efficient cattle. As such, molecular adaptations could have helped compensate for the lower feed intake in the most-efficient animals. The greater rate of VFA uptake in response to upregulation of VFA absorption in the ruminal epithelium of the most-efficient animals could have led to increases in intracellular proton (H^+^) load, hence, compromising intracellular pH (pH_i_) [55]. The Na^+^/H^+^ exchangers such as *SLC9A1*, which localize to the stratum granulosum of ruminal epithelium, play a central role in maintaining constant pH_i_ in rumen epithelial cells [56]. The greater expression of *SLC9A1* in the most-efficient steers could have been of physiologic importance because enhanced epithelial VFA uptake would have increased the intracellular H^+^ load. As such, the response in *SLC9A1* is suggestive of better functional capacity for the ruminal epithelium in the most-efficient cattle to maintain pH_i_. *ACO2* is an iron-sulfur enzyme that stimulates the conversion of citrate to isocitrate, an early step in the tricarboxylic acid (TCA) cycle [57]. Therefore, upregulation of *ACO2* in the ruminal epithelium of the most-efficient steers would be predicted to enhance activity of the TCA cycle, leading to greater energy production in ruminal epithelial cells.

#### Ketogenesis

The fact that the mRNA expression of *ACAT1*, *HMGCL, BDH1*, and *HMGCS2* was greater in the most-efficient animal suggests that epithelial butyrate utilization was more active in those animals, likely due to greater ruminal butyrate availability as shown by Guan et al. [58]. Greater butyrate production in the most-efficient animals is supported by the greater abundance of *M. elsdenii* and *E. ruminantium* in the rumen. Thus, the combined response in these genes suggests not only greater synthesis of hydroxybutyrate (BHBA) but potentially greater generation of ATP from metabolism of acetoacetyl-CoA. Because the reaction catalyzed by *ACAT1* is bi-directional, the greater expression of *ACAT1* in the most-efficient animals could have allowed for an increase in metabolism of acetyl-CoA generated from butyrate oxidation toward acetoacetyl-CoA [59]. The enzymes encoded by *HMGCS2* and *HMGCL* catalyze synthesis of hydroxyl-methyl-glutaryl-CoA and acetoacetate which can then be utilized by *BDH1* to generate BHBA [60, 61]. The fact that *HMGCS2* is transcriptionally-regulated suggests that most-efficient animals likely had an enhanced activity of the enzyme such that the main end-product BHBA was exported into the portal vein for utilization by peripheral tissues. Clearly, the upregulation of *ACAT1* also would have allowed for utilization of acetoacetyl-CoA for synthesis of ATP, i.e. re-circulation of BHBA back into ruminal epithelium through the bi-directional *BDH1* enzyme would have generated acetoacetyl-CoA followed by cleavage into 2 acetyl-CoA for further metabolism within the TCA cycle. The current study revealed no differences in *TECR* expression between RFI groups. However, Kong et al. [8] reported that *TECR* had greater expression in the ruminal epithelium of the most-efficient steers. This discrepancy between studies was likely driven by the greater number of animals (9 steers/group) used by Kong et al. [8], which may have allowed them to detect smaller differences in epithelial *TECR* expression between RFI groups.

#### Pyruvate metabolism

In coordination with the ketogenic genes, the upregulation of epithelial *LDHA*, *LDHB*, and *FFAR2* in the most-efficient cattle could have ensured that energy production is maintained in spite of lower feed intake. Lactate dehydrogenase A (*LDHA*) encodes a cytoplasmic enzyme catalyzing the conversion of pyruvate to lactate and NAD^+^ [62]. Lactate is then transported into the mitochondria for conversion into pyruvate and NADH via *LDHB*, promoting pyruvate oxidation in the TCA cycle and generation of reducing equivalents (e.g. NADH), which, in turn, can promote mitochondrial ATP synthesis and energy production [63]. The *PDHA1* gene encodes the subunit of the active site of the pyruvate dehydrogenase (PDH) enzyme that converts pyruvate to acetyl-CoA during glucose, lactate, or amino acid oxidation, and helps in the production of ATP [64]. The expression of *FFAR2* in ruminal epithelium of cattle is activated by luminal propionate concentration, hence, enhancing its metabolism to pyruvate via succinyl-CoA [65]. Thus, the greater *FFAR2* in most-efficient animals supports the possibility that higher amounts of ruminal propionate were produced, hence, upregulating *FFAR2*.

The lower expression of *PDHA1* in the most-efficient cattle supports the notion that there was greater production of ruminal butyrate. Previous reports detected an increase in epithelial *LDHA* and a decrease in *PDHA1* expression in beef and dairy cattle due to feed restriction or increased butyrate production in the rumen, respectively [22]. It was speculated that such responses would result in greater acetyl-CoA supply. Taken together, changes in the expression of *LDHA, LDHB, FFAR2,* and *PDHA1* in the ruminal epithelium of the most-efficient animals offers further support to the idea that those animals were able to more-efficiently capture energy from ruminal VFA.

#### Nuclear receptors

Among peroxisome proliferator-activated receptors measured in the current study, inducing *PPARD* could activate transcription of genes related to ketogenesis [66]. Therefore, we speculate that the upregulation of *PPARD* in the most-efficient animals was part of the transcriptional response that could have enhanced ketogenesis. This idea is supported in part by the upregulation of *ACAT1*, *HMGCL*, *BDH1*, and *HMGCS2*.

## Conclusions

An improvement of feed efficiency in the most-efficient cattle occurs in part through greater abundance of major bacterial species associated with fiber and non-fiber carbohydrate digestion that adhere to the ruminal solids-fraction, for example but not limited to *F. succinogenes, M. elsdenii* and *E. ruminantium*. The end-result is greater production of essential energy substrates for cattle (butyrate and propionate), which in turn activate metabolic pathways in ruminal epithelium to enhance VFA absorption and ketogenesis. Together, these adaptations would provide a greater energy supply to the host, which was partly reflected in the better carcass characteristics including HCW, KPH, and REA. The lack of association between RFI and sex on growth traits, carcass traits, and ruminal bacteria profiles underscore the independence of RFI. Overall, better feed efficiency as determined by RFI is at associated with unique adaptations in the ruminal microbiota and epithelium gene expression.

## Additional files

Additional file 1: **Table S1.** Primers sequence of targeted rumen bacterial species. **Table S2.** Metabolic pathway, gene symbol, and gene name of genes measured in rumen epithelium. **Table S3.** Primers sequence for rumen epithelial genes. **Table S4.** Real-time RT-PCR performance of genes measured in rumen epithelium. (DOCX 39 kb)

## Abbreviations

ADG: Average daily gain
BW: Body weight
DM: Dry matter
DMI: Dry matter intake
HCW: Hot carcass weight
KPH: Kidney, pelvic, and heart
NDF: Neutral detergent fiber
OM: Organic matter
REA: Ribeye area
RFI: Residual feed intake
TDN: Total digestible nutrients
