# Supplementary material for: Residual feed intake in beef cattle and its association with carcass traits, ruminal solid-fraction bacteria, and epithelium gene expression
Source: J Anim Sci Biotechnol. 2018 Sep 24;9:67. doi: 10.1186/s40104-018-0283-8 (PMC6151901; doi:10.1186/s40104-018-0283-8)
Supplement: Supplementary file 1 — Table S1. Primers sequence of targeted rumen bacterial species. Table S2. Metabolic pathway, gene symbol, and gene name of genes measured in rumen epithelium. Table S3. Primers sequence for rumen epithelial genes. Table S4. Real-time RT-PCR performance of genes measured in rumen epithelium. (DOCX 39 kb) [file 40104_2018_283_MOESM1_ESM.docx]

**ADDITIONAL FILE 1**

**Residual feed intake in beef cattle and its association with growth performance, carcass traits, ruminal solids-fraction bacteria, and ruminal epithelium gene expression**

Elolimy et al., 2018

**Table S1** Species-specific primers for the quantification of targeted rumen bacterial species in rumen solids-fraction of the most- (n=6) and the least- (n=6) efficient beef cattle by quantitative PCR assay after 70 d on RFI testing during the finishing period

| Target bacterial species |  | Primer sequence (5` - 3`) | Reference | qPCR efficiency^1^ (%) |
| --- | --- | --- | --- | --- |
| *Anaerovibrio lipolytica* | F:^2^  R:^3^ | GAAATGGATTCTAGTGGCAAACG  ACATCGGTCATGCGACCAA | [[22](#_ENREF_22)] | 104.89 |
| *Butyrivibrio proteoclasticus* | F:  R: | GGGCTTGCTTTGGAAACTGTT  CCCACCGATGTTCCTCCTAA | [[22](#_ENREF_22)] | 100.50 |
| *Eubacterium ruminantium* | F:  R: | CTCCCGAGACTGAGGAAGCTTG  GTCCATCTCACACCACCGGA | [[23](#_ENREF_23)] | 99.66 |
| *Fibrobacter succinogenes* | F:  R: | GCGGGTAGCAAACAGGATTAGA  CCCCCGGACACCCAGTAT | [[23](#_ENREF_23)] | 106.28 |
| *Megaspheara elsdenii* | F:  R: | AGATGGGGACAACAGCTGGA  CGAAAGCTCCGAAGAGCCT | [[23](#_ENREF_23)] | 102.21 |
| *Prevotella bryantii* | F:  R: | AGCGCAGGCCGTTTGG  GCTTCCTGTGCACTCAAGTCTGAC | [[23](#_ENREF_23)] | 105.35 |
| *Selenomonas ruminantium* | F:  R: | CAATAAGCATTCCGCCTGGG  TTCACTCAATGTCAAGCCCTGG | [[23](#_ENREF_23)] | 95.30 |
| *Succinimonas amylolytica* | F:  R: | CGTTGGGCGGTCATTTGAAAC  CCTGAGCGTCAGTTACTATCCAGA | [[24](#_ENREF_24)] | 98.84 |
| *Streptococcus bovis* | F:  R: | TTCCTAGAGATAGGAAGTTTCTTCGG  ATGATGGCAACTAACAATAGGGGT | [[23](#_ENREF_23)] | 96.45 |
| *Succinivibrio dextrinosolvens* | F:  R: | TAGGAGCTTGTGCGATAGTATGG  CTCACTATGTCAAGGTCAGGTAAGG | [[24](#_ENREF_24)] | 98.84 |
| Bacteria general 1 | F:  R: | GGATTAGATACCCTGGTAGT  CACGACACGAGCTGACG | [[18](#_ENREF_18)] | 94.92 |
| Bacteria general 2 | F:  R: | GTGSTGCAYGGYTGTCGTCA  ACGTCRTCCMCACCTTCCTC | [[17](#_ENREF_17)] | 98.03 |
| Bacteria general 3 | F: R: | CCTACGGGAGGCAGCAG  ATTACCGCGGCTGCTGG | [[20](#_ENREF_20)] | 101.78 |

^1^Measured efficiencies of the primers in the qPCR reactions

^2^F = forward primer

^3^R= reverse primer

**Table S2** Genes measured in rumen epithelium tissue of the most- (n=6) and the least- (n=6) efficient beef cattle by quantitative reverse transcription-PCR assay after 70 d on RFI testing during the finishing period

| Metabolic pathway and gene symbol | Gene name |
| --- | --- |
| VFA absorption |  |
| *SLC16A1* | Solute carrier family 16 member 1 |
| *SLC16A3* | Solute carrier family 16 member 3 |
| *SLC26A3* | Solute carrier family 26 member 3 |
| *MTCO2* | Mitochondrially Encoded Cytochrome C Oxidase II |
| *HCAR1* | Hydroxycarboxylic acid receptor 1 |
| *SLC9A1* | Solute carrier family 9 member A1 |
| *SLC9A2* | Solute carrier family 9 member A2 |
| *SLC9A3* | Solute carrier family 9 member A3 |
| *APPBP2* | Amyloid beta precursor protein binding protein 2 |
| *HIF1A* | Hypoxia inducible factor 1 alpha subunit |
| VFA metabolism |  |
| *ACSS1* | Acyl-CoA synthetase short-chain family member 1 |
| *ACSS2* | Acyl-CoA synthetase short-chain family member 2 |
| *ACO1* | Aconitase 1 |
| *ACO2* | Aconitase 2 |
| *PCCA* | Propionyl-CoA carboxylase alpha subunit |
| *SLC25A20* | Solute carrier family 25 member 20 |
| Ketogenesis |  |
| *ACADS* | Acyl-CoA dehydrogenase, C-2 to C-3 short chain |
| *ACAT1* | Acetyl-CoA acetyltransferase 1 |
| *HMGCL* | 3-Hydroxymethyl-3-methylglutaryl-CoA lyase |
| *BDH1* | 3-Hydroxybutyrate dehydrogenase 1 |
| *HMGCS2* | 3-Hydroxy-3-methylglutaryl-CoA synthase 2 |
| *TECR* | Trans-2,3-enoyl-CoA reductase |
| Pyruvate Metabolism |  |
| *LDHA* | Lactate dehydrogenase A |
| *LDHB* | Lactate dehydrogenase B |
| *PDHA1* | Pyruvate dehydrogenase alpha 1 |
| *PC* | Pyruvate carboxylase |
| Other metabolic pathways |  |
| *PPARA* | Peroxisome proliferator activated receptor alpha |
| *PPARG* | Peroxisome proliferator activated receptor gamma |
| *PPARD* | Peroxisome proliferator activated receptor delta |
| *FFAR2* | Free fatty acid receptor 2 |
| *RGS5* | Regulator of G protein signaling 5 |
| *NQO1* | NAD(P)H quinone dehydrogenase 1 |
| Immune/inflammation-response |  |
| *TLR2* | Toll like receptor 2 |
| *TLR4* | Toll like receptor 4 |
| Internal controls |  |
| *CMTM6* | CKLF like MARVEL transmembrane domain containing 6 |
| *ERC1* | ELKS/RAB6-interacting/CAST family member 1 |
| *MRPL39* | Mitochondrial ribosomal protein L39 |

**Table S3** Oligonucleotide primer sequences for genes measured in rumen epithelium tissue of the most- (n=6) and the least- (n=6) efficient beef cattle by quantitative reverse transcription-PCR assay after 70 d on RFI testing during the finishing period

| Gene symbol^1^ |  | Primer sequence (5`- 3`) | Product size (bp) | GenBank accession number |
| --- | --- | --- | --- | --- |
| *SLC16A1* | F:  R: | CCTGTGGGACTGAAGGGTAAAT  ATGATTCCCACAGAAATGTCCAGTAT | 110 | NM_001037319.1 |
| *SLC16A3* | F:  R: | GTCAACGCTCGTATTTATTTCACAA  AAAACACTGATCGGGTGATGATC | 101 | NM_001109980.2 |
| *SLC26A3* | F:  R: | CACACCGATCCATTTTCACTTTT  CCAGGATAACCAAGGATGTCACA | 100 | NM_001083676.1 |
| *MTCO2* | F:  R: | CATGGGCTGTGCCCTCTCTA  TGACCCGCAAATTTCTGAGC | 122 | NM_001034707.2 |
| *HCAR1* | F:  R: | ACAGGTCGTGCTGTCTCATC  AATAGTGCTCGGCTTCCAGG | 149 | NM_001145234.1 |
| *SLC9A1* | F:^2^  R:^3^ | TCAGGCATCATGGCACTCATT  ATGAGGGTTTCACTGACGCT | 131 | NM_174833.2 |
| *SLC9A2* | F:  R: | GAGACCGTTGACGTGTTTGCT  TGGTAAACGCCGCTATAAAGC | 100 | XM_019969840.1 |
| *SLC9A3* | F:  R: | CCCGGCAGGAGTACAAACAT  TTGGCCGACTTGAAGGACTC | 124 | NM_001192154.1 |
| *APPBP2* | F:  R: | TCTCCGCTGTGGTGGATAAC  CGTCCCTGTTGGTATAGCTTGT | 103 | XM_015467947.1 |
| *HIF1A* | F:  R: | CACACAGAAATGGCCTTGTGA  ATGGCCTGTGCAGTGAAGTA | 146 | NM_174339.3 |
| *ACSS1* | F:  R: | GGATCAACGATGCCCAATGC  GTTGTCTGTCCTGTGAGCCA | 149 | NM_174746.2 |
| *ACSS2* | F:  R: | GGCGAATGCCTCTACTGCTT  GGCCAATCTTTTCTCTAATCTGCTT | 100 | NM_001105339.1 |
| *ACO1* | F:  R: | GATGTTAGGGGCAGGCTTGT  GGTAAGGCATGACTCCGCTT | 137 | NM_001075591.1 |
| *ACO2* | F:  R: | AGTACCATGTGGCCTCAGTC  GTTCAGCCGTTTGCGGAC | 125 | NM_173977.3 |
| *PCCA* | F:  R: | GGGTTACCCTCTCAGGCACAA  CAACAGACGGCAAACCAAAA | 110 | NM_001083509.1 |
| *SLC25A20* | F:  R: | TCATGCGAGATGTTCCAGCC  CCAGTTGAAGATCCCCGCAA | 146 | NM_001077936.2 |
| *ACADS* | F:  R: | GAGTGTCAACAATTCCCTCTACTTAG  GGTTCACTGAGGGCAAAGCA | 124 | NM_001034401.2 |
| *ACAT1* | F:  R: | GTCGGAGATCATGCCTGTGTT  TGATACATAACTTCGCTCCGCATA | 113 | NM_001046075.1 |
| *HMGCL* | F:  R: | AATCTTGAAGGCAGCTCGGG  TTCTTGGTGACCTCAGCGAC | 117 | NM_001075132.1 |
| *BDH1* | F:  R: | CTGCGAAGCGCCAGGTTATC  CCTCCTGGTGGGTTCCCAAA | 91 | NM_001034600.2 |
| *HMGCS2* | F:  R: | TTACGGGCCCTGGACAAAT  GCACATCATCGAGAGTGAAAGG | 100 | NM_001045883.1 |
| *TECR* | F:  R: | CCATGCCCTTGCGAAACATC  GGCAGATCACAAAGATGGCGA | 150 | NM_001034748.2 |
| *LDHA* | F:  R: | GCCTGTATGGAGTGGAGTGAATG  GTGAACCGCTTTCCACTGTTC | 100 | NM_174099.2 |
| *LDHB* | F:  R: | TTGTCTTCGTGCAGCCCTTA  TTCTTCTGCAACTGGTGCAATC | 100 | NM_174100.2 |
| *PDHA1* | F:  R: | GAGTGAGTTACCGTACCCGAGAA  ACTGGCGAGATTGCTGTTCA | 101 | NM_001101046.2 |
| *PC* | F:  R: | GCAAGGTCCACGTGACTAAGG  GGCAGCACAGTGTCCTGAAG | 124 | NM_177946.4 |
| *PPARA* | F:  R: | CATAACGCGATTCGTTTTGGA  CGCGGTTTCGGAATCTTCT | 102 | NM_001034036.1 |
| *PPARG* | F:  R: | GAGCCCAAGTTCGAGTTTGC  GGCGGTCTCCACTGAGAATAAT | 100 | NM_181024.2 |
| *PPARD* | F:  R: | CATCATTGAGCCCAAGTTCGA  CGATGAAGAGAGCCAGGTCACT | 80 | NM_001083636.1 |
| *FFAR2* | F:  R: | ATCGCCTGGGTCATGTCTTT  AGCAGGTGGTCTCATTCTCCTTT | 100 | NM_001163784.1 |
| *RGS5* | F:  R: | CTGCAGAACAATTATGGACTGGC  TCAGCCATCTTGACAGGGGA | 128 | NM_001034707.2 |
| *NQO1* | F:  R: | AGTTTCCCCTGCAGTGGTTT  AGCACTGCCTTCTTATTCCGA | 133 | NM_001034535.1 |
| *TLR2* | F:  R: | CCATGTCTGGAGAGGGTGTT  GGGGACACAAAACAGCACTT | 102 | NM_174197.2 |
| *TLR4* | F:  R: | GCTGTTTGACCAGTCTGATTGC  GGGCTGAAGTAACAACAAGAGGAA | 102 | NM_174198.6 |
| *CMTM6* | F:  R: | TTATCACTCTGGGCACAGGAC  ACATCCCAAACACGATTGCAG | 105 | NM_001035066.1 |
| *ERC1* | F:  R: | GCTCTCCTGGAACTGTCGTC  CGATTTTGTGTCTGCTGCTTCA | 110 | NM_001205419.1 |
| *MRPL39* | F:  R: | AGGTTCTCTTTTGTTGGCATCC  TTGGTCAGAGCCCCAGAAGT | 101 | NM017446 |

^1^Details of the genes are reported in Table S2

^2^F = forward primer

^3^R= reverse primer

**Table S4** Quantitative PCR performance of selected ruminal epithelium genes in the most- (n=6) and the least- (n=6) efficient beef cattle after 70 d on RFI testing during the finishing period

| Gene symbol^1^ | Median Ct^2^ | Median ∆Ct^3^ | Slope^4^ | (R^2^)^5^ | Efficiency^6^ | Relative mRNA abundance^7^ | 1/E∆Ct | (%)^8^ |
| --- | --- | --- | --- | --- | --- | --- | --- | --- |
| *SLC16A1* | 20.23 | -2.79 | -3.16 | 0.994 | 2.074 | 7.6310 | 0.016482 | 1.64819 |
| *SLC16A3* | 28.97 | 5.92 | -3.17 | 0.994 | 2.068 | 0.0136 | 0.000029 | 0.00293 |
| *SLC26A3* | 21.44 | -1.47 | -3.21 | 0.991 | 2.048 | 2.8595 | 0.006176 | 0.61761 |
| *MTCO2* | 15.77 | -7.38 | -3.19 | 0.997 | 2.059 | 205.9239 | 0.444765 | 44.47652 |
| *HCAR1* | 31.49 | 8.57 | -3.30 | 0.993 | 2.009 | 0.0025 | 0.000005 | 0.00055 |
| *SLC9A1* | 24.67 | 1.59 | -3.18 | 0.987 | 2.063 | 0.3162 | 0.000683 | 0.06829 |
| *SLC9A2* | 20.97 | -2.07 | -3.12 | 0.997 | 2.091 | 4.6202 | 0.009979 | 0.99789 |
| *SLC9A3* | 19.89 | -3.14 | -2.99 | 0.995 | 2.158 | 11.2216 | 0.024237 | 2.42370 |
| *APPBP2* | 20.93 | -2.15 | -3.35 | 0.993 | 1.988 | 4.3936 | 0.009490 | 0.94896 |
| *HIF1A* | 23.95 | 0.93 | -3.32 | 0.995 | 2.000 | 0.5261 | 0.001136 | 0.11362 |
| *ACSS1* | 28.64 | 5.48 | -3.19 | 0.992 | 2.057 | 0.0192 | 0.000042 | 0.00416 |
| *ACSS2* | 24.41 | 1.46 | -3.33 | 0.989 | 1.997 | 0.3638 | 0.000786 | 0.07858 |
| *ACO1* | 23.27 | 0.31 | -3.31 | 0.996 | 2.004 | 0.8058 | 0.001740 | 0.17404 |
| *ACO2* | 21.36 | -1.68 | -3.29 | 0.992 | 2.012 | 3.2421 | 0.007002 | 0.70024 |
| *PCCA* | 23.00 | 0.05 | -3.28 | 0.993 | 2.020 | 0.9634 | 0.002081 | 0.20809 |
| *SLC25A20* | 24.09 | 1.14 | -3.35 | 0.990 | 1.989 | 0.4582 | 0.000990 | 0.09896 |
| *ACADS* | 18.77 | -4.33 | -3.36 | 0.996 | 1.986 | 19.5355 | 0.042194 | 4.21938 |
| *ACAT1* | 21.46 | -1.67 | -3.15 | 0.991 | 2.080 | 3.4086 | 0.007362 | 0.73621 |
| *HMGCL* | 21.89 | -1.05 | -3.15 | 0.998 | 2.080 | 2.1541 | 0.004652 | 0.46525 |
| *BDH1* | 20.99 | -1.91 | -3.26 | 0.991 | 2.027 | 3.8590 | 0.008335 | 0.83349 |
| *HMGCS2* | 16.10 | -6.89 | -3.13 | 0.998 | 2.086 | 158.7072 | 0.342784 | 34.27841 |
| *TECR* | 20.62 | -2.56 | -3.14 | 0.991 | 2.084 | 6.5665 | 0.014183 | 1.41826 |
| *LDHA* | 19.08 | -3.95 | -3.32 | 0.997 | 2.000 | 15.4893 | 0.033455 | 3.34547 |
| *LDHB* | 21.15 | -1.84 | -3.18 | 0.992 | 2.065 | 3.7887 | 0.008183 | 0.81830 |
| *PDHA1* | 23.23 | 0.14 | -3.19 | 0.986 | 2.056 | 0.9037 | 0.001952 | 0.19518 |
| *PC* | 27.75 | 4.72 | -3.24 | 0.992 | 2.036 | 0.0349 | 0.000075 | 0.00755 |
| *PPARA* | 25.33 | 2.28 | -3.01 | 0.983 | 2.150 | 0.1740 | 0.000376 | 0.03759 |
| *PPARG* | 22.33 | -0.74 | -3.22 | 0.993 | 2.043 | 1.7030 | 0.003678 | 0.36783 |
| *PPARD* | 22.87 | -0.14 | -3.23 | 0.995 | 2.042 | 1.1032 | 0.002383 | 0.23827 |
| *FFAR2* | 33.92 | 10.70 | -3.01 | 0.978 | 2.152 | 0.0003 | 0.000001 | 0.00006 |
| *RGS5* | 22.19 | -0.77 | -3.20 | 0.994 | 2.055 | 1.7472 | 0.003774 | 0.37736 |
| *NQO1* | 24.12 | 1.27 | -3.18 | 0.992 | 2.062 | 0.3990 | 0.000862 | 0.08618 |
| *TLR2* | 27.24 | 4.09 | -3.32 | 0.991 | 2.002 | 0.0585 | 0.000126 | 0.01264 |
| *TLR4* | 32.68 | 9.69 | -3.28 | 0.998 | 2.017 | 0.0011 | 0.000002 | 0.00024 |

^1^Details of the genes are reported in Table 3

^2^ The median is calculated considering rumen fluid- and solids- fractions for all animals

^3^ The median of ∆Ct is calculated as [Ct gene – geometrical mean of Ct internal controls] for rumen fluid- and solids-fractions for all animals

^4^ Slope of the 6-point standard curve

^5^ R^2^ stands for the coefficient of determination of the standard curve

^6^ Efficiency of amplification is calculated as [10^(-1 / Slope)^]

^7^ Relative mRNA abundance = 1/ Efficiency ^Median ∆Ct^

^8^1/E∆Ct (%) = (relative mRNA abundance of specific gene/∑relative mRNA abundance of all genes) × 100
